# Engineering ergothioneine production in *Yarrowia lipolytica*

**DOI:** 10.1002/1873-3468.14239

**Published:** 2021-12-05

**Authors:** Steven A. van der Hoek, Matej Rusnák, Irene Hjorth Jacobsen, José L. Martínez, Douglas B. Kell, Irina Borodina

**Affiliations:** ^1^ The Novo Nordisk Foundation Center for Biosustainability Technical University of Denmark Kongens Lyngby Denmark; ^2^ Department of Biotechnology and Biomedicine Technical University of Denmark Kongens Lyngby Denmark; ^3^ Department of Biochemistry and Systems Biology Institute of Systems, Molecular and Integrative Biology University of Liverpool UK

**Keywords:** ergothioneine, metabolic engineering, nutraceutical, phosphate‐limitation, *Yarrowia lipolytica*

## Abstract

Ergothioneine is a naturally occurring antioxidant that has shown potential in ameliorating neurodegenerative and cardiovascular diseases. In this study, we investigated the potential of the Crabtree‐negative, oleaginous yeast *Yarrowia lipolytica* as an alternative host for ergothioneine production. We expressed the biosynthetic enzymes *EGT1* from *Neurospora crassa* and *EGT2* from *Claviceps purpurea* to obtain 158 mg·L^−1^ of ergothioneine in small‐scale cultivation, with an additional copy of each gene improving the titer to 205 mg·L^−1^. The effect of phosphate limitation on ergothioneine production was studied, and finally, a phosphate‐limited fed‐batch fermentation in 1 L bioreactors yielded 1.63 ± 0.04 g·L^−1^ ergothioneine in 220 h, corresponding to an overall volumetric productivity of 7.41 mg·L^−1^·h^−1^, showing that *Y*. *lipolytica* is a promising host for ergothioneine production.

## Graphical abstract

Here, we report that the oleaginous yeast *Yarrowia lipolytica* is a viable host to produce the antioxidant ergothioneine. *Yarrowia lipolytica* could reach ergothioneine production levels comparable to or exceeding those reported in other hosts, such as *Escherichia coli* and *Saccharomyces cerevisiae*, by genomic integration of the ergothioneine biosynthetic pathway and fed‐batch fermentation on mineral medium.

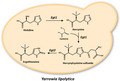

## Abbreviations

**CDW**, cell dry weight

**ERG**, ergothioneine

**MES**, 2‐(*N*‐morpholino)ethanesulfonic acid

**SAM**, *S*‐adenosylmethionine

The amino acid‐derived nutraceutical ergothioneine (ERG) has recently gained much scientific interest for its potential application in preventing or treating neurodegenerative and cardiovascular diseases [1, 2]. Current scientific understanding attributes its capacity in ameliorating disease to its potent antioxidant properties [3], as oxidative damage is commonly present in chronic, inflammatory diseases [4]. While ERG is ubiquitous in nature [5], it is only produced by fungi and bacteria. To date, no higher eukaryotes have been reported to biosynthesize ERG.

**Fig. 1:**
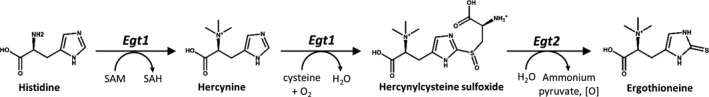
The fungal biosynthesis pathway of ERG. [O], oxidation; SAH, *S*‐adenosylhomocysteine.

Ergothioneine is biosynthesized from the precursors histidine, cysteine, and *S*‐adenosylmethionine (SAM; Fig. 1). In the fungal pathway, histidine is methylated three times by Egt1 to form hercynine before the same enzyme attaches cysteine to generate hercynylcysteine sulfoxide. The β‐lyase enzyme Egt2 then dissociates ammonium pyruvate from the intermediate, and a subsequent reduction of the sulfur produces ERG. The bacterial pathway (Fig. S1) has the disadvantage of using five enzymes compared to two, and ATP is used to form other pathway intermediates that include glutamate, which has to be cleaved off in later steps.

Both pathways have previously been used to produce ERG in cell factories (Table 1). *Aspergillus oryzae* produced 231 mg of ERG·kg^−1^ solid media after integrating multiple copies of the *EGT1* and *EGT2* genes of *Neurospora crassa* [6]. *Escherichia* *coli* produced 24 mg·L^−1^ of ERG after expression of the *Mycobacterium* *smegmatis egtBCDE* genes and the addition of amino acid precursors to the medium [7]. Further metabolic engineering for improved cysteine and SAM biosynthesis enhanced the production in *E. coli* to 1.3 g·L^−1^ in 216 h when precursors were supplemented to the cells [8]. Previously, we screened various combinations of fungal and bacterial ERG biosynthesis enzymes to find the optimal combination for ERG production in *Saccharomyces cerevisiae*. Two copies of the *N. crassa EGT1* and the *Claviceps purpurea EGT2* gene produced 598 mg·L^−1^ of ERG in an 84‐h fed‐batch cultivation where additional amino acid precursors were fed to the cells [9].

**Table 1:** ERG production using engineered microbial cell factories.

Host organism	Genetic engineering	ERG production	Medium	Cultivation conditions	Reference
*Aspergillus oryzae*	Multicopy integration of *EGT1* and *EGT2* genes from *Neurospora crassa* under the *amyB* promoter	231.0 ± 1.1 mg·kg^−1^ solid medium in 120 h	Solid medium containing Nanatsuboshi rice and adenine	50 mL petri dish at 30 °C	[1]
*Escherichia coli*	BW25113 parent strain (high l‐Cys producer), expression of *egtBCDE* genes from *Mycobacterium smegmatis* under the *tac* promoter on high‐copy, episomal plasmids	24 ± 4 mg·L^−1^ in 72 h	M9Y medium supplemented with l‐His, l‐Met, ferric sulfate, and sodium thiosulfate. Induction with IPTG	50 mL medium in 200 mL Erlenmeyer flask at 30 °C and 200 r.p.m.	[2]
*Escherichia coli*	BW25113 parent strain (high l‐Cys producer), expression of *egtABCDE* genes from *M. smegmatis* under the *tac* promoter, and expression of feedback‐insensitive *cysE* (T167A), feedback‐insensitive *serA* (T410stop), and wild‐type *ydeE* genes from *E. coli* under the *ompA* promoter on high‐copy, episomal plasmids, and deletion of the native *metJ* gene	1311 ± 275 mg·L^−1^ in 216 h	Batch phase – Medium containing glucose, salts, tryptone, yeast extract, l‐Met, and ammonium ferric citrate Fed‐batch phase – A 400 g·L^−1^ glucose solution was fed (0.65 L), and additional feeding of a solution containing with l‐His, l‐Met, sodium thiosulfate (0.25 L). One time addition of pyridoxine, ammonium ferric citrate, and IPTG	Fed‐batch fermentation in a 3 L jar fermenter from Sanki Seiki, 30 °C and 490 r.p.m., aeration 1 L·min^−1^, pH 6.9–7.0	[3]
*Saccharomyces cerevisiae*	CEN.PK113‐7D parent strain, integration of two copies of the *EGT1* gene of *N. crassa* and the *EGT2* gene of *Claviceps purpurea* under the *TEF1* promoter	598 ± 18 mg·L^−1^ in 84 h	Batch phase – 0.5 L Mineral medium supplemented with l‐Arg, l‐His, l‐Met, and pyridoxine Fed‐batch phase – 0.5 L feeding medium contained 415 g/L glucose, salts, vitamins, trace metals, l‐Arg, l‐His, l‐Met, and pyridoxine	Fed‐batch fermentation in 1 L Sartorius bioreactors, 30 °C and 500 r.p.m., aeration 0.5 L·min^−1^, pH 5.0	[4]
*Yarrowia lipolytica*	W29 strain with integrated Cas9 and deletion of *ku70*, integration of two copies of the *EGT1* gene of *N. crassa* and the *EGT2* gene of *C. purpurea* under the control of the *TEFintron* and the *GPD* promoter	1.63 ± 0.04 g·L^−1^ in 220 h	Batch phase – 0.5 L mineral medium supplemented with pyridoxine Fed‐batch phase – 0.5 L feeding medium containing 730 g·L^−1^ glucose, salts, vitamins, trace metals, pyridoxine	Fed‐batch fermentation in 1 L Sartorius bioreactors, 30 °C and 800–1200 r.p.m., aeration 0.5–1.0 L·min^−1^, pH 5.0	This study

*Yarrowia lipolytica* is an oleaginous yeast capable of accumulating high amounts of storage lipids. In some wild‐type strains, lipid content can reach 20–40% of dry weight, while in engineered strains, lipid content can exceed 90% [10]. Lipid production requires cytosolic acetyl‐CoA, malonyl‐CoA, and NADPH [11]. As these precursors are also required for isoprenoids, polyketides, and other metabolites, *Y. lipolytica* is also a promising cell factory for the production of such metabolites [11, 12, 13, 14, 15]. *Yarrowia* *lipolytica* has been engineered for the production of β‐carotene [14], β‐farnesene, limonene, valencene, squalene, 2,3‐oxidosqualene [12], aromatic amino acids [15], and resveratrol [13]. Additionally, *Y. lipolytica* is used for the production of lipases, β‐mannases, laccases, amylases, and proteases [16].

In contrast to *S. cerevisiae*, *Y. lipolytica* is a Crabtree‐negative yeast, so it does not have an extensive overflow metabolism in the presence of sugar excess [17] and is therefore much easier to ferment at large scale. The aim of this study was to explore *Y. lipolytica* as the host for the production of ERG.

## Material and methods

### Strains and chemicals

The *Y*. *lipolytica* strain ST6512 [18] was used as the background strain for metabolic engineering. *Escherichia* *coli* DH5α was used for all cloning procedures, propagation, and storing of plasmids. ERG (catalog # E7521‐25MG, ≥ 98% purity) was bought from Sigma‐Aldrich (St. Louis, MO, USA). Synthetic genes were ordered through the GeneArt Gene Synthesis service of Thermo Fisher Scientific (Waltham, MA, USA) or the custom gene synthesis service of IDT (Newark, NJ, USA). Sequencing results were obtained through Eurofins Genomics (Ebersberg, Germany) using their Mix2Seq kit.

### Media

Mineral medium consisted of 20 g·L^−1^ of glucose, 7.5 g·L^−1^ of (NH_4_)_2_SO_4_, 14.4 g·L^−1^ of KH_2_PO_4_, 0.5 g·L^−1^ of MgSO_4_.7H_2_O, 2 mL·L^−1^ of trace metal solution, and 1 mL·L^−1^ of vitamin solution, adjusted to pH 6.0 using 2 m of NaOH. Mineral medium without phosphate was prepared using the same recipe, but KH_2_PO_4_ was substituted with the same concentration of 2‐(*N*‐morpholino)ethanesulfonic acid (MES) hydrate. High glucose mineral medium without phosphate consisted of 50 g·L^−1^ of glucose, 20 g·L^−1^ of (NH_4_)_2_SO_4_, 30 g·L^−1^ of MES hydrate, 1.25 g·L^−1^ of MgSO_4_.7H_2_O, 5 mL·L^−1^ of trace metal solution, and 2.5 mL·L^−1^ of vitamin solution, adjusted to pH 6.0 using 2 m of NaOH.

Trace metal solution consisted of 4.5 g·L^−1^ of CaCl_2_.2H_2_O, 4.5 g·L^−1^ of ZnSO_4_.7H_2_O, 3 g·L^−1^ of FeSO_4_.7H_2_O, 1 g·L^−1^ of H_3_BO_3_, 1 g·L^−1^ of MnCl_2_.4H_2_O, 0.4 g·L^−1^ of Na_2_MoO_4_.2H_2_O, 0.3 g·L^−1^ of CoCl_2_.6H_2_O, 0.1 g·L^−1^ of CuSO_4_.5H_2_O, 0.1 g·L^−1^ of KI, and 15 g·L^−1^ of EDTA. Vitamin solution consisted of 50 mg·L^−1^ of biotin, 200 mg·L^−1^ of *p*‐aminobenzoic acid, 1 g·L^−1^ of nicotinic acid, 1 g·L^−1^ of calcium pantothenate, 1 g·L^−1^ of pyridoxine HCl, 1 g·L^−1^ of thiamine HCl, and 25 g·L^−1^ of myo‐inositol.

### Cloning

Strain construction for the integrations in *Y. lipolytica* was performed using the EasyCloneYALI method [18]. After transformation with plasmids, *E. coli* was grown on LB plates with 100 mg·L^−1^ of ampicillin. For the selection of *Y. lipolytica* strains after modification with Cas9 plus gRNA, YPD plates supplemented with 250 mg·L^−1^ of nourseothricin were used. Strains were checked for correct genetic modification by colony PCR. A list of the used genes, primers, biobricks, plasmids, and strains can be found in Tables S1–S6.

### Small‐scale cultivation conditions

For checking initial ERG production, a single colony of the respective strains was inoculated in 5 mL of mineral medium in a 13‐mL preculture tube and cultured two times overnight at 30 °C and 250 r.p.m. ERG production of the strains was tested in mineral medium by cultivating for 48 h at 30 °C and 250 r.p.m. Precultures for phosphate‐limited experiments were made by inoculating a single colony of ST10264 in 5 mL of mineral medium without phosphate, supplemented with 0.04 g·L^−1^ of KH_2_PO_4_, and culturing the strain three times overnight at 30 °C and 250 r.p.m. The correlation of biomass to phosphate concentration was performed by inoculating strain ST10264 in mineral medium without phosphate with either 20 g·L^−1^ of glucose or 50 g·L^−1^ of glucose, supplemented with various concentrations of KH_2_PO_4_, at OD_600_ = 0.1 in 2 mL of medium in 24 deep‐well plates. The strain was then cultivated for 72 h at 30 °C and 250 r.p.m. The OD_600_ values were determined using the NanoPhotometer Pearl (Implen, Munich, Germany). Phosphate‐limited ERG production was determined by inoculating 50 mL of high glucose mineral medium without phosphate, supplemented with 0.04 g·L^−1^ KH_2_PO_4_, in 250‐mL baffled shake flask at OD_600_ = 0.2 using inoculum from phosphate‐limited precultures. The strain was cultivated for 56 h at 30 °C and 250 r.p.m.

### HPLC analyses

Ergothioneine was quantified by HPLC, similar to our previous work [9]. The ERG extraction was performed similar to Ref. [19]. ERG concentrations were determined by taking a 1 mL sample of the cultivation broth and immediately boiling the sample at 94 °C for 10 min, with subsequent vortexing at 1600 r.p.m. for 30 min using a DVX‐2500 Multi‐Tube Vortexer from VWR. The vortexed samples were centrifuged at 10 000 ***g*** for 5 min, and the supernatant was taken and analyzed using HPLC. If storage was necessary, samples were stored at −20 °C. For HPLC analysis, the Dionex Ultimate 3000 HPLC system with the analysis software chromeleon (Thermo Fisher Scientific) was used. Samples were run on a Cortecs UPLC T3 reversed‐phase column (particle size 1.6 µm, pore size 120 Å, 2.1 × 150 mm). The flow rate was 0.3 mL·min^−1^, starting with 2.5 min of 0.1% formic acid, going up to 70% acetonitrile, 30% 0.1% formic acid at 3 min for 0.5 min, after which 100% 0.1% formic acid was run for 4–9 min. ERG was detected at a wavelength of 254 nm. For analysis of bioreactor samples, we additionally quantified glucose, ethanol, pyruvate, and acetate concentrations by HPLC as described [20].

### Testing biomass accumulation during a phosphate‐limited fed‐batch fermentation in a bioreactor

Batch phase medium consisted of 20 g·L^−1^ of glucose, 16 g·L^−1^ of (NH_4_)_2_SO_4_, 15 g·L^−1^ of MES hydrate, 0.34 g·L^−1^ of KH_2_PO_4_, 2 g·L^−1^ of MgSO_4_.7H_2_O, 40 mg·L^−1^ of pyridoxine hydrochloride, 2 mL·L^−1^ of trace metal solution, and 1 mL·L^−1^ of vitamin solution. Feed medium consisted of 700 g·L^−1^ of glucose, 96 g·L^−1^ of (NH_4_)_2_SO_4_, 15 g·L^−1^ of MES hydrate, 16 g·L^−1^ of MgSO_4_.7H_2_O, 0.5 g·L^−1^ of pyridoxine hydrochloride, 20 mL·L^−1^ of trace metal solution, and 10 mL·L^−1^ of vitamin solution.

A single colony from a YPD plate with ST10264 colonies was used to inoculate 5 mL of mineral medium with 20 g·L^−1^ of glucose but without phosphate, supplemented with 0.04 g·L^−1^ of KH_2_PO_4_ in a 13‐mL preculture tube. The tube was incubated at 30 °C and 250 r.p.m. two times overnight. Two milliliters of this preculture were used to inoculate 98 mL of mineral medium with 20 g·L^−1^ of glucose but without phosphate, supplemented with 0.04 g·L^−1^ of KH_2_PO_4_, in a 500‐mL baffled shake flask. The shake flask was then incubated two times overnight at 30 °C and 250 r.p.m. The culture was centrifuged at 3000 ***g*** for 5 min, the supernatant was decanted, and the pellet was resuspended in 50 mL of sterile MilliQ water. The precultured cells were then used to inoculate 0.7 L batch phase medium in a single 1‐L Sartorius bioreactor. The starting OD_600_ was 0.2. The stirring rate was set at 800 r.p.m., the airflow was set at 0.5 SLPM, the temperature was kept at 30 °C, and pH was maintained at pH 5.0 using 2 m of KOH and 2 m of H_2_SO_4_. The stirring was controlled by the level of dissolved oxygen in the solution. If it dropped below 40%, the stirring was increased up to 1200 r.p.m. The feeding was started after 40 h when all the glucose was consumed. The airflow was set to 1.0 SLPM during the feeding. The starting feed rate at 40 h was 2.6 g·h^−1^ (2.0 mL·h^−1^). At 64 h, the feed rate was increased to 3.9 g·h^−1^ (3.0 mL·h^−1^). At 76 h, the feed rate was decreased to 3.3 g·h^−1^ (2.5 mL·h^−1^). At 124 h, the feed rate was decreased to 2.6 g·h^−1^ (2.0 mL·h^−1^) until the feeding was stopped at 172 h. Antifoam was added as necessary.

### Phosphate‐limited fed‐batch fermentation in bioreactors

Batch phase medium consisted of 40 g·L^−1^ of glucose, 16 g·L^−1^ of (NH_4_)_2_SO_4_, 0.83 g·L^−1^ of KH_2_PO_4_, 2 g·L^−1^ of MgSO_4_.7H_2_O, 40 mg·L^−1^ of pyridoxine hydrochloride, 4 mL·L^−1^ of trace metal solution and 2 mL·L^−1^ of vitamin solution. Feed medium consisted of 730 g·L^−1^ of glucose, 136 g·L^−1^ of (NH_4_)_2_SO_4_, 16 g·L^−1^ of MgSO_4_.7H_2_O, 1 g·L^−1^ of pyridoxine hydrochloride, 20 mL·L^−1^ of trace metal solution, and 10 mL·L^−1^ of vitamin solution.

A single colony from a YPD plate with ST10264 colonies was used to inoculate 5 mL of mineral medium with 20 g·L^−1^ of glucose but without phosphate, supplemented with 0.04 g·L^−1^ of KH_2_PO_4_ in a 13‐mL preculture tube. The tube was incubated at 30 °C and 250 r.p.m. two times overnight. Two milliliters of this preculture were used to inoculated 98 mL of mineral medium with 20 g·L^−1^ of glucose but without phosphate, supplemented with 0.04 g·L^−1^ of KH_2_PO_4_, in a 500‐mL baffled shake flask. The shake flask was then incubated two times overnight at 30 °C and 250 r.p.m. The culture was centrifuged at 3000 ***g*** for 5 min, the supernatant was decanted, and the pellet was resuspended in 50 mL of sterile MilliQ water. The precultured cells were then used to inoculate 0.6 L of batch phase medium in 1‐L Sartorius bioreactors in triplicate. The starting OD_600_ was 0.2. The temperature was kept at 30 °C, and pH was maintained at pH 5.0 using 2 m of KOH. The dissolved oxygen level controlled a cascade for the stirring and airflow. If the dissolved oxygen level dropped below 40%, the stirring was first increased from 800 to a maximum of 1200 r.p.m., followed by the airflow from 1.0 SLPM to a maximum of 1.5 SLPM. The feeding was started after 40 h when all the glucose was consumed. The starting feed rate at 40 h was 2.6 g·h^−1^ (2.0 mL·h^−1^). At 64 h, the feed rate was increased to 3.3 g·h^−1^ (2.5 mL·h^−1^). At 76 h, the feed rate was increased to 4.0 g·h^−1^ (3.0 mL·h^−1^). At 88 h, the feed rate was increased to 4.7 g·h^−1^ (3.5 mL·h^−1^). Finally, at 100 h, the feed rate was increased to 5.3 g·h^−1^ (4.0 mL·h^−1^) until the feeding was stopped at 160 h. Antifoam was added as necessary.

## Results and Discussion

### Integration of the ergothioneine biosynthesis pathway

**Fig. 2:**
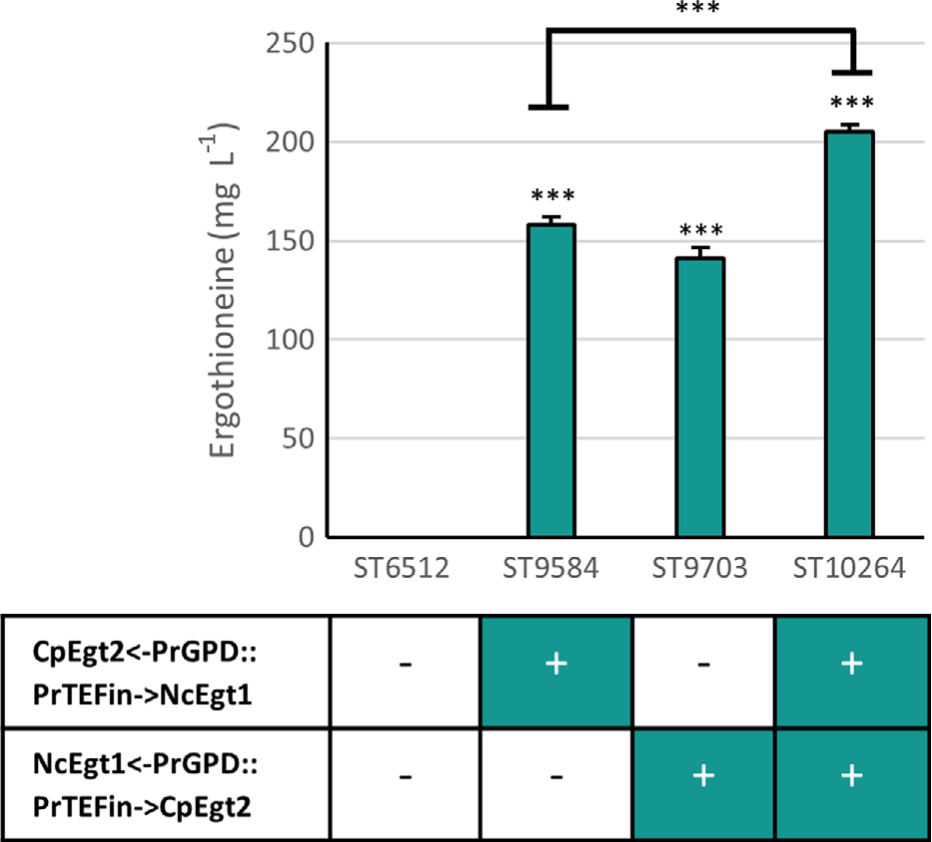
ERG production titers of engineered *Yarrowia* *lipolytica* strains (*n* = 3, error bars signify SD). The Nc*EGT1* and Cp*EGT2* genes were integrated in single copies under different promoters, or in two copies. A two‐tailed Student's *t*‐test was used to determine if the difference was significant between the control strain ST6512 and the strain with the integrated ERG biosynthesis pathway (***P‐value < 0.0005).

To implement ERG biosynthesis in *Y. lipolytica*, we chose the combination of enzymes that performed optimally in *S. cerevisiae*, viz. the *EGT1* gene from *N. crassa* (Genbank accession XP_956324.3) and the *EGT2* gene from *C. purpurea* (Genbank accession CCE33140.1) [9]. The genes were codon‐optimized for *Y. lipolytica* expression and placed under the control of either TEFintron promoter or GPD promoter in the ST6512 strain. ST6512 is derived from *Y*. *lipolytica* W29 by integrating the *cas9* gene and knocking out of the *ku70* gene to reduce the nonhomologous end joining [18]. While both TEFintron and GPD promoters are strong constitutive promoters, TEFintron promoter is several‐fold stronger [18]. The expression of a single copy of the pathway allowed *Y. lipolytica* to produce 141 or 158 mg·L^−1^ ERG, depending on the promoter choice (Fig. 2). The higher titer was obtained for the strain expressing NcEgt1 under the control of TEFintron promoter, which is in line with the results in *S. cerevisiae*, where an additional copy of *N. crassa EGT1* improved the production of ERG, but not an extra copy of *C. purpurea EGT2* [9]. Interestingly, these titers, obtained in a simple small‐scale batch cultivation, were 3‐fold higher than the titers obtained for an analogous *S. cerevisiae* strain cultivated in simulated fed‐batch medium and 9‐fold higher than *S. cerevisiae* titers in batch medium [9]. As the next step, we integrated another copy of both *EGT1* and *EGT2* genes to obtain strain ST10264, which produced 205 mg·L^−1^ of ERG (Fig. 2). Comparatively, the ERG titer in *E. coli* expressing the bacterial pathway on high‐copy number plasmids was approximately 22 mg·L^−1^ [8]. These results show that *Y. lipolytica* is a suitable host for the production of ERG.

### Phosphate limits biomass accumulation but not ergothioneine production in small‐scale cultivations

Biomass concentration should be carefully considered for a successful fed‐batch cultivation of the ERG‐producing ST10264 strain. *Yarrowia* *lipolytica* grows to cell densities as high as 80–120 g·L^−1^ during fed‐batch cultivation depending on the substrate, as evidenced in [13, 21, 22]. As the biosynthesis pathway for ERG requires oxygen, high cell density in fed‐batch conditions can be disadvantageous for the production of ERG. The solution was to limit the final biomass by means other than the typical carbon limitation in fed‐batch cultivations. Nitrogen limitation is often used in *Y. lipolytica* cultivations, but nitrogen limitation triggers downregulation of amino acid synthesis. The carbon flux is then redirected to lipid metabolism [23]. Since ERG is derived from the amino acids histidine and cysteine, and the amino acid‐derived SAM, nitrogen limitation should not be used to limit the biomass concentration in fed‐batch cultivation.

**Fig. 3:**
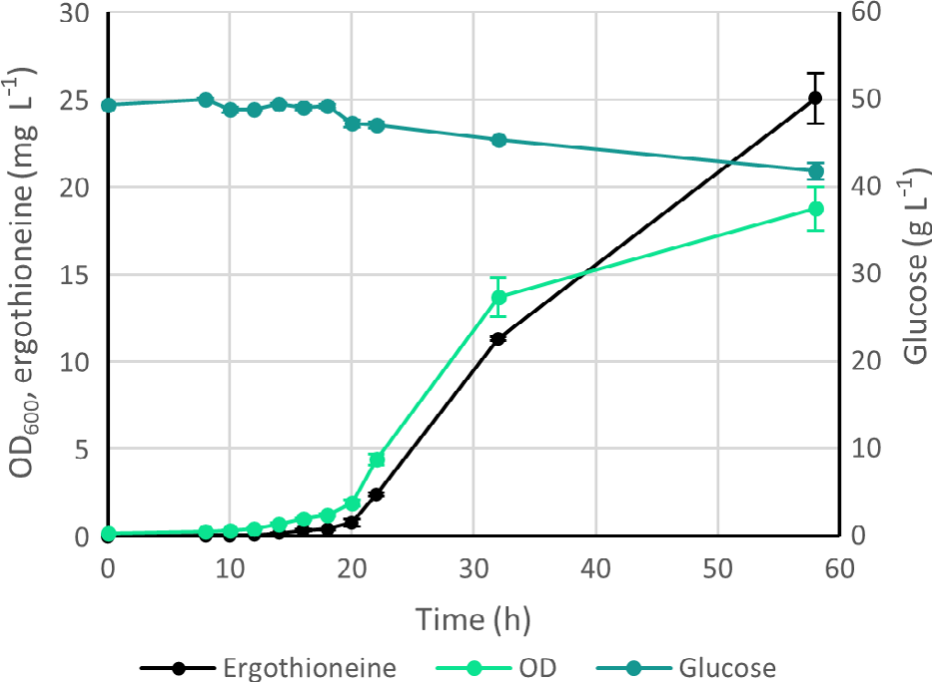
The effect of phosphate limitation on the growth and production of the ERG‐producing *Yarrowia* *lipolytica* strain ST10264 when cultivated in 250‐mL baffled shake flasks in medium containing 0.04 g·L^−1^ of KH_2_PO_4_ (*n* = 3, error bars signify SD).

Therefore, it was investigated if the biomass of strain ST10264 could be limited by the phosphate concentration instead (Fig. S2). Phosphate is typically used to buffer the medium, so we used MES hydrate for pH buffering instead. We tested two glucose concentrations (20 or 50 g·L^−1^) and different KH_2_PO_4_ concentrations (0–0.3 g·L^−1^) for cellular growth in 24‐deep‐well plates (Fig. S2). In parallel, we cultivated the same strain in shake flasks with 0.04 g·L^−1^ of KH_2_PO_4_ and 50 g·L^−1^ of glucose to determine if ERG would be produced after the strain stopped growing. Indeed, while the OD_600_ increased by only ca. 30% in the second half of cultivation, ERG concentration doubled during the same period (Fig. 3B). These experiments indicated that phosphate limitation is a viable strategy for ERG fermentation.

### Producing ergothioneine using phosphate‐limited fed‐batch fermentation

When limiting the biomass using the phosphate concentration in 24‐deep‐well plates, the OD_600_ values increased by 4–5 units per 0.01 g·L^−1^ of KH_2_PO_4_ in the KH_2_PO_4_ concentration range of 0.01 g·L^−1^ to 0.08 g·L^−1^ (Fig. S2), corresponding to an increase of ca 0.48–0.60 g·L^−1^ cell dry weight (CDW) per 0.01 g·L^−1^ of KH_2_PO_4_. We then designed an initial fermentation experiment to confirm that the KH_2_PO_4_ concentration limited the biomass to the same level in a bioreactor. Therefore, we added 0.24 g of KH_2_PO_4_ per 1 L of final volume of fed‐batch cultivation in a bioreactor, meaning that 0.34 g·L^−1^ of KH_2_PO_4_ was added to the starting volume of 0.7 L, to reach 15–20 g·L^−1^ CDW during the fermentation. The results of this experimental phosphate‐limited fed‐batch fermentation in a single bioreactor are shown in Fig. S3. After the initial batch phase of 40 h, the glucose feeding was started at a rate of 1.4 g·h^−1^ (2.0 mL·h^−1^ feed solution) and was adjusted throughout the fermentation to ensure that the added glucose was not entirely consumed by the cells.

The strain produced ERG throughout the fed‐batch fermentation, with the highest productivity of 10.07 mg·L^−1^·h^−1^ ERG when phosphate was not limiting the growth. However, when phosphate became limiting, the productivity dropped to 2.77 mg·L^−1^·h^−1^ ERG. Curiously, strain ST10264 reached 32–33 g·L^−1^ CDW already at 76 h, and the biomass concentration did not increase until 100 h. Even though the biomass concentration was higher than the aimed 15–20 g·L^−1^ of CDW, the dissolved oxygen level did not drop below 40%, meaning no oxygen limitation was observed. Therefore, 60 mg of KH_2_PO_4_ was added to the bioreactor at 100 h to study its effect on biomass concentration, and the additional KH_2_PO_4_ increased the CDW of ST10264 to 40 g·L^−1^. Furthermore, the productivity increased to 5.01–6.59 mg·L^−1^·h^−1^ ERG upon addition of potassium phosphate, as calculated using either the lower ERG titers or higher ERG titers between 136 and 208 h. Surprisingly, the strain generated approximately 1.33 g·L^−1^ of CDW per 0.01 g·L^−1^ of KH_2_PO_4_, more than double the level of biomass compared to the experiment in 24‐deep‐well plates.

**Fig. 4:**
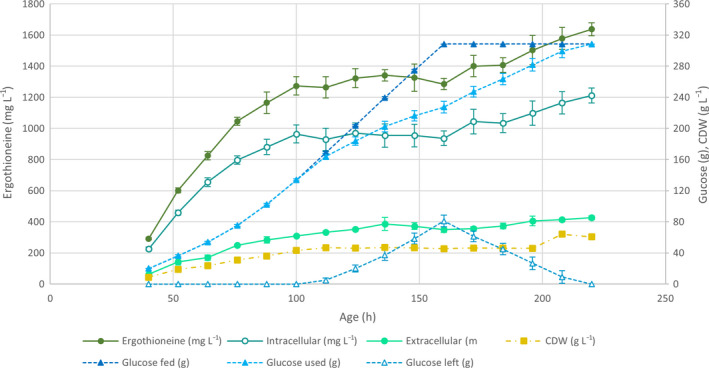
Phosphate‐limited fed‐batch fermentation of ST10264 in 1 L bioreactors. The total, extracellular and intracellular ERG levels are represented by lines with circular markers. The amount of glucose that was fed to the strain, used by the strain and left in the reactor is represented by lines with triangular markers. The CDW is represented by a line with square markers. Average values and standard deviations are presented in the graph (*n* = 3, error bars signify SD).

From the results of this trial fermentation, we estimated that a biomass concentration of approximately 60 g·L^−1^ of CDW would not cause oxygen limitation issues. Hence, we added 0.5 g of KH_2_PO_4_ per 1 L of the final volume of fed‐batch cultivation in bioreactors, equivalent to 0.83 g·L^−1^ of KH_2_PO_4_ in the starting volume of 0.6 L. After inoculation at OD_600_ = 0.2, the cells were grown for 40 h in the batch phase to accumulate enough biomass to start feeding. The dissolved oxygen level was controlled through a cascade of stirring (800–1200 r.p.m.) and airflow (1.0–1.5 SLPM). The feeding was started after the batch phase concluded at 2.6 g·h^−1^ (2.0 mL·h^−1^) and kept at that level for 24 h. After that, the feed rate was increased by 0.65 g·h^−1^ (0.5 mL·h^−1^) every 12 h until glucose started accumulating in the medium. The feed rate reached a maximum of 5.3 g·h^−1^ (4.0 mL·h^−1^) between 100 and 160 h, after which the feeding was stopped. Figure 4 details the results of the phosphate‐limited fed‐batch fermentation in 1 L bioreactors performed in triplicate.

Overall, strain ST10264 produced 1637 ± 41 mg·L^−1^ of ERG after 220 h, out of which 26% was extracellular and 74% was intracellular. The overall productivity was 7.41 ± 0.19 mg·L^−1^·h^−1^ ERG. Both the overall titer and productivity were higher than obtained with *E. coli*, which produced 1.3 g·L^−1^ ERG with a productivity of 6.02 mg·L^−1^·h^−1^ [8], and *S. cerevisiae* producing 598 mg·L^−1^ ERG at a rate of 7.12 mg·L^−1^·h^−1^ [9]. However, ERG yield was highest at 16.36 mg·L^−1^·h^−1^ until 100 h, when phosphate became limiting, and glucose started accumulating in the medium. After phosphate became limiting, the ERG titers remained approximately the same between 100 and 160 h, while the volume inside the bioreactor increased at a rate of 4.0 mL·h^−1^. The overall productivity at 100 h was 12.73 mg·L^−1^·h^−1^, which dropped to 8.03 mg·L^−1^·h^−1^ at 160 h. Thus, we estimated the productivity between 100 and 160 h to be 4.70 mg·L^−1^·h^−1^ to account for the volume increase in this timeframe. After the feeding stopped and excess glucose was consumed by the cells, the ERG titer increased from 1285 to 1637 mg·L^−1^ between 160 and 220 h, showing a productivity of 5.87 mg·L^−1^·h^−1^ in this period.

The final biomass concentration of the fermentation was 60.6 g·L^−1^ of CDW, close to the target of 60 g·L^−1^ of CDW of the experimental design. However, the biomass concentration was 45–46 g·L^−1^ CDW until 196 h. The lower than expected biomass concentration until 196 h could have been caused by increased use of phosphate for general metabolite production in the early fed‐batch phase, as the ERG productivity until 100 h was 16.36 mg·L^−1^·h^−1^, compared to 10.07 mg·L^−1^·h^−1^ in the early fed‐batch phase of the fermentation with a lower amount of phosphate. The sudden increase in biomass concentration occurred when excess glucose in the medium reached below 10 g·L^−1^ glucose and became limiting. We hypothesize that the lower glucose concentration initiated a change in metabolism that allowed the generation of additional biomass.

The production of ERG by *Y. lipolytica* had another notable advantage over *E. coli* and *S. cerevisiae*. In both the *E. coli* and the *S. cerevisiae* studies, the fermentations were supplemented with additional amino acid precursors to improve titers (Table 1) [8, 9]. Furthermore, *E. coli* was additionally supplemented with ammonium ferric citrate. The fermentation media used here only contained an additional 1 g·L^−1^ of pyridoxine, a cofactor for the CpEgt2 enzyme. Pyridoxine was supplemented to the media in the *E. coli* and *S. cerevisiae* studies at 10 and 0.5 g·L^−1^, respectively [8, 9]. Thus, using *Y. lipolytica* as a host also improved the cost‐effectiveness of ERG production.

In conclusion, we integrated the ERG biosynthesis pathway into oleaginous yeast *Y. lipolytica*. Phosphate limitation was a viable strategy to limit biomass accumulation in the bioreactors. ERG titer reached 1.63 ± 0.04 g·L^−1^ after 220 h of fermentation in mineral medium with glucose as the only carbon source.

## Conflict of interest

SH, DBK, and IB are named inventors on a European Patent application covering parts of the work described above.

## Author contributions

IB and DBK conceived the study; IB and JLM supervised the study; SAH, IHJ, and JLM designed the experiments; SAH, IHJ, and MR performed the experiments and data analysis; SAH and IB wrote the manuscript; SAH, IB, DBK, IHJ, and JLM reviewed and revised the manuscript; IB and JLM provided resources for the study; IB acquired funding for the study.

## Supporting information


**Fig. S1**. Bacterial ergothioneine biosynthesis pathway.
**Fig. S2**. The effect of phosphate limitation on the growth of the ergothioneine‐producing *Yarrowia lipolytica* strain ST10264.
**Fig. S3**. Ergothioneine production and biomass accumulation of ST10264 under phosphate‐limited fed‐batch conditions in a single 1 L bioreactor with an initial amount of 240 mg KH_2_PO_4_.
**Table S1**. List with genes and their DNA sequences used in this paper.
**Table S2**. List of primers used in this study for cloning purposes.
**Table S3**. List of primers used in this study for sequencing.
**Table S4**. List of biobricks used in this study.
**Table S5**. List of plasmids used in this study, made by USER cloning.
**Table S6**. List of strains used in this study.

## Data Availability

The data that support the findings of this study are available in Figs 1, 2, 3, 4 and the supplementary material of this article.
