# Supplementary material for: Engineering ergothioneine production in Yarrowia lipolytica
Source: FEBS Lett. 2021 Dec 5;596(10):1356–64. doi: 10.1002/1873-3468.14239 (PMC9299812; doi:10.1002/1873-3468.14239)
Supplement: Supplementary file 1 — Fig. S1. Bacterial ergothioneine biosynthesis pathway. Fig. S2. The effect of phosphate limitation on the growth of the ergothioneine‐producing Yarrowia lipolytica strain ST10264. Fig. S3. Ergothioneine production and biomass accumulation of ST10264 under phosphate‐limited fed‐batch conditions in a single 1 L bioreactor with an initial amount of 240 mg KH2PO4. Table S1. List with genes and their DNA sequences used in this paper. Table S2. List of primers used in this study for cloning purposes. Table S3. List of primers used in this study for sequencing. Table S4. List of biobricks used in this study. Table S5. List of plasmids used in this study, made by USER cloning. Table S6. List of strains used in this study. [file FEB2-596-1356-s001.docx]

**Fig. S1:** Bacterial ergothioneine biosynthesis pathway.


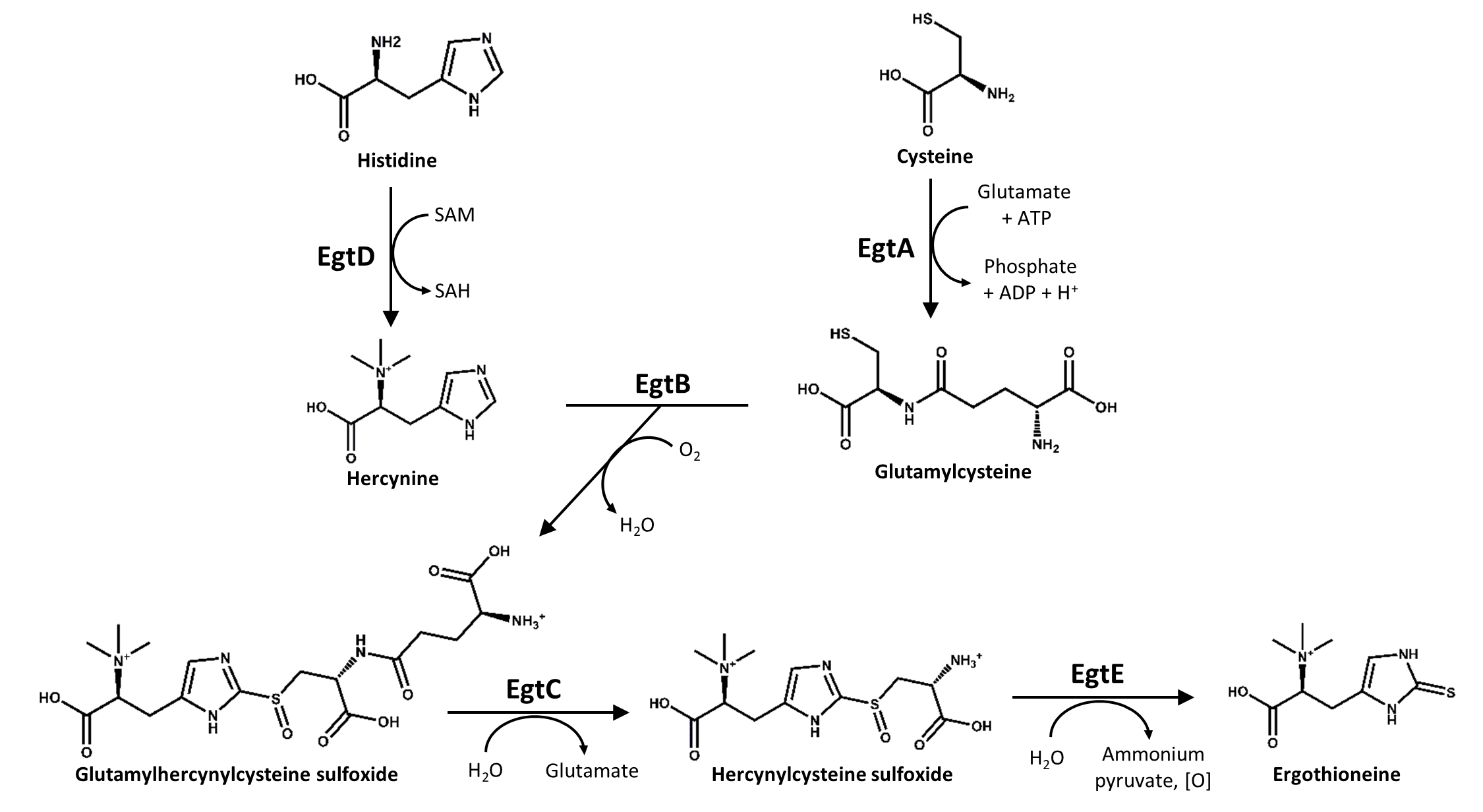


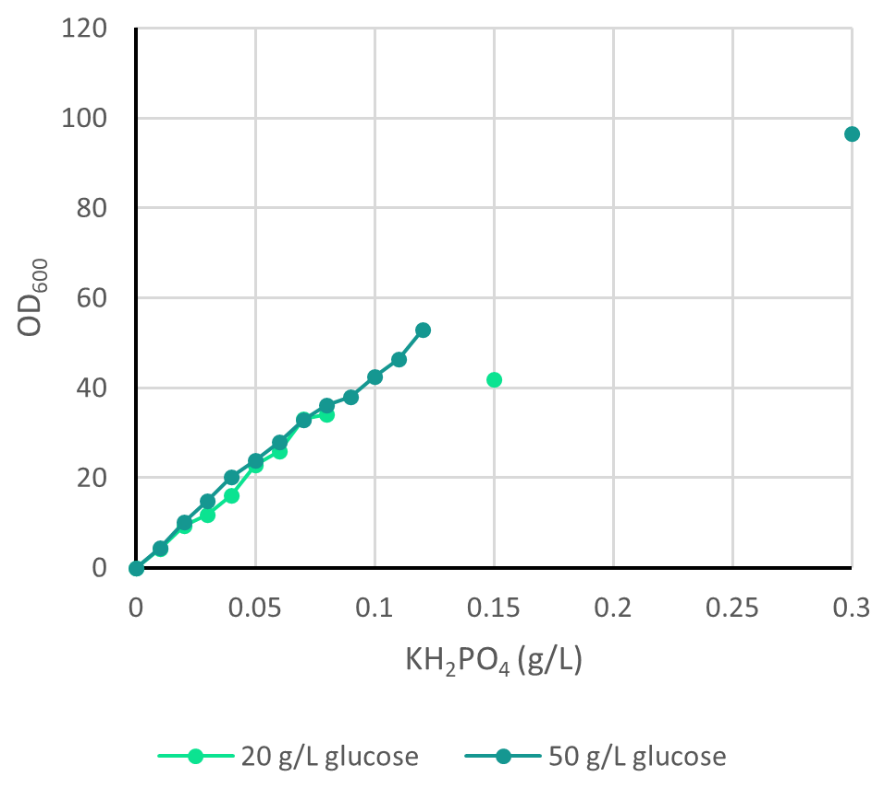


**Fig. S2:** The effect of phosphate limitation on the growth of the ergothioneine-producing Y. lipolytica strain ST10264.


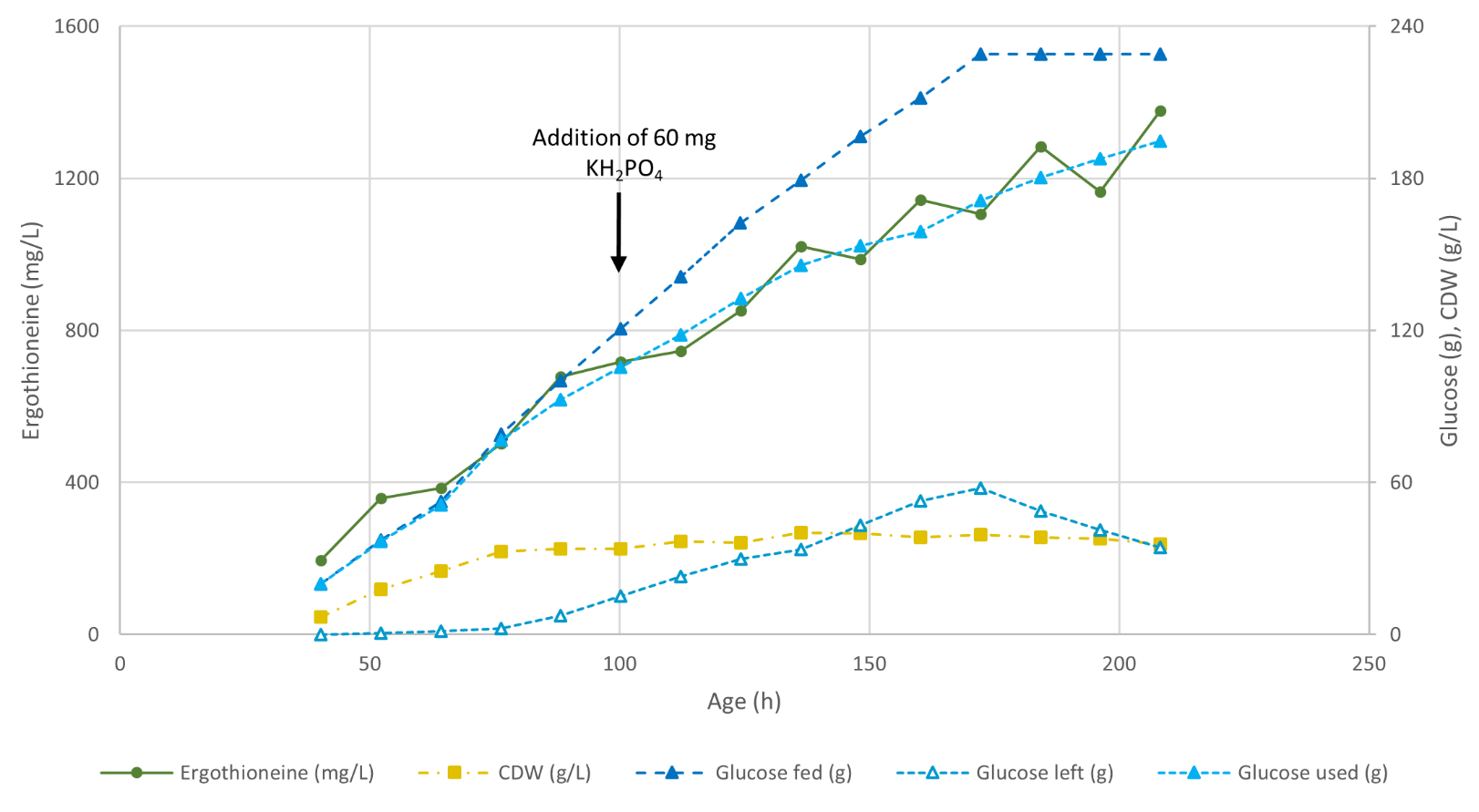


**Fig. S3:** Ergothioneine production and biomass accumulation of ST10264 under phosphate-limited fed-batch conditions in a single 1 L bioreactor with an initial amount of 240 mg KH_2_PO_4_.

**Table S1:** List with genes and their DNA sequences used in this paper.

| **Gene** | **DNA sequence** | **Source** |
| --- | --- | --- |
| NcEgt1 | ATGCCCTCTGCCGAGTCTATGACCCCTTCTTCGGCCCTGGGCCAGCTGAAGGCCACCGGCCAGCACGTCCTGTCTAAGCTGCAGCAGCAGACCTCTAACGCCGACATCATCGACATCCGACGAGTGGCCGTCGAGATCAACCTCAAGACCGAGATCACCTCTATGTTCCGACCTAAGGACGGACCCCGACAGCTGCCCACTCTGCTGCTCTACAACGAGCGAGGCCTGCAGCTGTTCGAGCGAATCACCTACCTGGAAGAGTACTACCTGACCAACGACGAGATCAAGATCCTGACCAAGCACGCTACCGAGATGGCCTCTTTCATCCCCTCTGGCGCCATGATCATCGAGCTTGGCTCTGGCAACCTGCGAAAGGTGAACCTGCTGCTCGAGGCCCTGGACAACGCCGGCAAGGCCATCGACTACTACGCCCTGGACCTGTCTCGAGAGGAACTCGAGCGAACCCTGGCTCAGGTGCCCTCTTACAAGCACGTGAAGTGCCACGGCCTGCTGGGCACCTACGACGACGGCCGAGACTGGCTGAAGGCTCCCGAGAACATCAACAAGCAGAAGTGCATCCTGCACCTGGGATCTTCTATCGGCAACTTCAACCGATCTGACGCCGCCACCTTCCTGAAGGGCTTCACCGACGTGCTCGGCCCCAACGACAAGATGCTGATCGGCGTGGACGCCTGCAACGACCCCGCTCGAGTGTACCACGCCTACAACGATAAGGTGGGCATCACCCACGAGTTCATCCTGAACGGCCTGCGAAACGCCAACGAGATCATCGGCGAGACTGCCTTCATCGAAGGCGACTGGCGAGTGATCGGCGAGTACGTGTACGACGAGGAAGGCGGCCGACACCAGGCCTTCTACGCTCCCACTCGAGACACCATGGTCATGGGCGAGCTGATCCGATCTCACGACCGAATCCAGATCGAGCAGTCTCTGAAGTACTCTAAGGAAGAGTCTGAGCGACTCTGGTCTACCGCCGGACTCGAGCAGGTTTCTGAGTGGACCTACGGCAACGAGTACGGCCTGCACCTCCTGGCCAAGTCTCGAATGTCTTTCTCTCTGATCCCCTCCGTGTACGCCCGATCTGCCCTGCCTACTCTGGACGACTGGGAAGCCCTGTGGGCCACCTGGGACGTCGTGACCCGACAGATGCTGCCCCAAGAGGAACTGCTCGAGAAGCCCATCAAGCTCCGAAACGCCTGCATCTTCTACCTGGGACACATCCCCACCTTTCTGGACATCCAGCTGACCAAGACCACCAAGCAGGCTCCCTCTGAGCCCGCTCACTTCTGCAAGATCTTCGAGCGAGGAATCGACCCCGACGTGGACAACCCCGAGCTGTGTCACGCCCACTCCGAGATTCCCGATGAGTGGCCTCCTGTGGAAGAGATTCTGACCTACCAAGAGACTGTGCGATCTCGACTGCGAGGACTGTACGCCCACGGAATCGCCAACATTCCCCGAAACGTGGGCCGAGCCATCTGGGTGGGCTTCGAGCACGAGCTGATGCACATCGAGACTCTGCTGTACATGATGCTGCAGTCTGACAAGACCCTGATTCCTACTCACATCCCTCGACCTGACTTCGACAAGCTGGCCCGAAAGGCCGAGTCCGAGCGAGTGCCCAACCAGTGGTTCAAGATCCCCGCTCAAGAGATCACCATCGGCCTGGACGACCCTGAGGACGGCTCTGACATTAACAAGCACTACGGCTGGGACAACGAGAAGCCTCCTCGACGAGTGCAGGTCGCCGCCTTTCAGGCCCAGGGACGACCCATCACCAACGAGGAATACGCCCAGTACCTCCTCGAGAAGAACATTGACAAGCTGCCTGCCTCTTGGGCTCGACTGGATAACGAGAACATTTCTAACGGCACCACCAACTCTGTGTCTGGCCACCACTCTAACCGAACCTCTAAGCAGCAGCTGCCCTCTTCGTTCCTGGAAAAGACCGCCGTGCGAACCGTGTACGGACTGGTGCCCCTGAAGCACGCTCTGGACTGGCCCGTGTTCGCCTCTTACGACGAGCTGGCTGGCTGCGCCGCCTACATGGGCGGACGAATTCCCACCTTCGAGGAAACCCGATCTATCTACGCCTACGCCGACGCTCTCAAGAAGAAGAAGGAAGCCGAGCGACAGCTGGGCCGAACCGTGCCTGCCGTGAACGCCCACCTCACCAACAACGGCGTCGAGATTACCCCTCCTTCGTCGCCCTCGTCTGAGACTCCCGCCGAGTCCTCTTCTCCCTCCGACTCTAACACCACTCTGATTACCACCGAGGACCTGTTCTCTGACCTGGACGGCGCCAACGTGGGCTTCCACAACTGGCACCCCATGCCTATCACCTCCAAGGGCAACACCCTGGTCGGCCAGGGCGAGCTTGGAGGCGTGTGGGAGTGGACTTCTTCTGTGCTGCGAAAGTGGGAGGGATTCGAGCCCATGGAACTGTACCCCGGCTACACCGCCGACTTTTTCGACGAGAAGCACAACATCGTGCTCGGAGGATCTTGGGCTACCCATCCTCGAATCGCCGGACGAAAGTCCTTCGTGAACTGGTATCAGCGAAACTACCCCTACGCCTGGGTGGGAGCCCGAGTGGTGCGAGATCTGTAA | Synthetic gene codon-optimized for *Y. lipolytica*  Genbank accession XP_956324.3 |
| CpEgt2 | ATGGGTCTGCTGGAAGGTGAGGAACTGGTGCTGCGAGGTCGGGGACAAGGCGGTGAACCACGACCCGAGAGAGAGCCCGAACTGAAGCTCGAACACGTTCCTGAGCGAGCTCCCGATGGTGAACCTGAGACTGAGGGTCAGCTAGGCCCCCGAAAGGAGCCTGAGCACAAGCTCGAGGCTGAGTCCGAGCCACTCCAGGAGACTCCCCAGCGAGAGGTGCTCGCCTTCGGTCGAGCTTGGAAGTCTGAGTTCCTCTTCGACCCCGCATGGCGAAACCTCAACCACGGCTCCTTTGGCACCTACCCTCTGTACATCCGAGACAAGCTGCGAGCTTACCAGGATCAGGCCGAGGCTCGACCCGACCACTTCATCCGATACGAGGAGAGTAAGCTTCTGCACCGATCCCGAGCCGCTGTGGCTAAGATCGTTAACGCTCCCCTCGACACCGTGGTGTTCGTTGGTAACGCCACGGAGGGTGTGAACACCGTTCTGCGAAACCTGCGATGGGACTCTCTGGAGAAGGGCGGCCAGAAGGATGTCATCCTCTCTTTCTCCACCGTCTACGAGGCCTGTGGTAATGCTGCCGACTACATCGTCGAGTACTTCGCTGGTAAGGTCGAGCATAGGACCATTGAGCTGGAGTACCCCGTGGAGGACGCTGACGTGATCGCCGCACTGCGAGGCGCCGCCACTCAGGTCGCCCGAGAGGGCAAGCGAGCCCGGCTCGCCATGATGGATGTGGTCACCTCCCGACCTGGCGTCGTCTTTCCCTGGGAAGCCGCCGTTCGAGTCTGTCGGGAGCTGGGTATCCTTTCCCTCGTCGACGGCGCTCAGGGTGTTGGCATGGTGCGACTGGACCTCACCGCTGCTGACCCCGACTTTTTCGTTTCCAACTGCCACAAGTGGCTGCTTGTCCCCCGAGGCTGTGCTATGCTTTACACCCCCGCTAGAACCCAGTGTCTGCTGCGAACTGCTCTGGCCACTTCCCACGGTTACGTTCCTCCTTCCGCTGCTCCCGCTCCTCCTGGGTCCAAGTCCCGATATGTCGCCAACTTCGAGTTCGTCGGTACTCGAGATAACGGACCCTACCTGTGTGTCGCCGACGCCATTGCCTGGCGAGAGCGAGTTTGTGGCGGAGAGGAGAACATCCTGCGGTACCTGTGGGCCCTTAACAAGAAAGGTATTCGAATTGTCGCTCGTGCTCTGGGAACTACTCATCTTGATAACGAGACCGAGACTCTGACAAACTGTGCCATGGGCAACGTCGCCCTGCCCATGAGAGTCGATGACGAGGACGCCTCCACCGCCCTGGACGCAGCTCCGTCGGCCGCTATTGCTGCTCCTGACGTTGTTGTTGCTCGAGAGAATGTGGCCCTGGTGGATAAGTGGATGCGCGAACGACTCTTCGACGACTACAAGACCTTCATGACCCTGTTCGTTATGCAGGACCGATACTGGGTCCGACTGTCCGCCCAAATTTACCTCGATGAGCAGGATTACGAGGCTGCTGGAGACATCCTCAAGGCCCTGTGTGAGCGAATTCGACGACGAGAGTACCTGGTCCCCCAGCCCGTCGAGTAA | Synthetic gene codon-optimized for *Y. lipolytica*  Genbank accession CCE33140.1 |

**Table S2:** List of primers used in this study for cloning purposes.

| **ID** | **Name** | **Sequence 5´to 3´** |
| --- | --- | --- |
| PR-23965 | PrGPD-R | ACCTGCACUTGTTGATGTGTGTTTAATTCAAGAATGAAT |
| PR-23966 | PrTEF1in-F | AGCTACTGAUAGAGACCGGGTTGGCGGC |
| PR-25255 | NcEgt1-fwd_TEFin_Gene2 | ACTTTTTGCAGTACUAACCGCAGCCCTCTGCCGAGTCTATGAC |
| PR-25256 | NcEgt1_rev_Gene2 | CACGCGAUTTACAGATCTCGCACCACTCG |
| PR-25257 | CpEgt2_fwd_Gene1 | AGTGCAGGUGCCACAATGGGTCTGCTGGAAGGTGA |
| PR-25258 | CpEgt2_rev_Gene1 | CGTGCGAUTTACTCGACGGGCTGGGG |
| PR-26229 | NcEgt1-Gene1_fw | AGTGCAGGUGCCACAATGCCCTCTGCCGAGTC |
| PR-26230 | NcEgt1-Gene1_rv | CGTGCGAUTTACAGATCTCGCACCACTCGG |
| PR-26231 | CpEgt2_TEFin-Gene2_fw | ACTTTTTGCAGTACUAACCGCAGGGTCTGCTGGAAGGTGAGG |
| PR-26232 | CpEgt2-Gene2_rv | CACGCGAUTTACTCGACGGGCTGGG |

**Table S3:** List of primers used in this study for sequencing.

| **ID** | **Name** | **Sequence 5´to 3´** | **Used for** |
| --- | --- | --- | --- |
| PR-14617 | Gene1_check_rev | TATCCCTGTGTTGAATC | pCfB9216,  pCfB9324,  pCfB10020 |
| PR-14619 | Gene2_check_rev | TATCGACCCAGTTAGC | pCfB9216,  pCfB9324,  pCfB10020 |
| PR-25255 | NcEgt1_fwd_TEFin_Gene2 | ACTTTTTGCAGTACUAACCGCAGCCCTCTGCCGAGTCTATGAC | pCfB9216,  pCfB10020 |
| PR-26231 | CpEgt2_TEFin-Gene2_fw | ACTTTTTGCAGTACUAACCGCAGGGTCTGCTGGAAGGTGAGG | pCfB9324 |
| PR-26237 | CpEgt2_Yl_seq1 | ACTCGAAGTTGGCGAC | pCfB9216,  pCfB9324,  pCfB10020 |
| PR-26238 | CpEgt2_Yl_seq2 | AGCGTTAACGATCTTAGCC | pCfB9216,  pCfB9324,  pCfB10020 |
| PR-26239 | PrGPD-Tefint_seq1 | TAACCTGATGTGGTGCAAAA | pCfB9216,  pCfB9324,  pCfB10020 |
| PR-26240 | PrGPD-Tefint_seq2 | ACGAAATGCCTACCCC | pCfB9216,  pCfB9324,  pCfB10020 |
| PR-26241 | NcEgt1_Yl_seq1 | AACGACAAGATGCTGATCG | pCfB9216,  pCfB9324,  pCfB10020 |
| PR-26242 | NcEgt1_Yl_seq2 | ATCTTCGAGCGAGGAATC | pCfB9216,  pCfB9324,  pCfB10020 |
| PR-26243 | NcEgt1_Yl_seq3 | ATCTTGATCTCGTCGTTGG | pCfB9216,  pCfB9324,  pCfB10020 |

**Table S4:** List of biobricks used in this study.

| **ID** | **Name** | **Primers** | **Template** |
| --- | --- | --- | --- |
| BB3907 | <-PrGPD::PrTEFin-> | PR-23965,  PR-23966 | pCfB8811 |
| BB4204 | NcEgt1-Yl_Gene2 | PR-25255,  PR-25256 | pCfB10864 (synthetic NcEgt1 gene) |
| BB4205 | CpEgt2-Yl_Gene1 | PR-25257,  PR-25258 | pCfB10865 (synthetic CpEgt2 gene) |
| BB4332 | NcEgt1-Yl_Gene1 | PR-26229,  PR-26230 | pCfB10864 (synthetic NcEgt1 gene) |
| BB4333 | CpEgt2-Yl_Gene2 | PR-26231,  PR-26232 | pCfB10865 (synthetic CpEgt2 gene) |

**Table S5:** List of plasmids used in this study, made by USER cloning.

| **ID** | **Name** | **Description** | **Template** | **Biobricks** | **Source** |
| --- | --- | --- | --- | --- | --- |
| **Basic vectors** | | | | | |
| pCfB8811 | Int C_2 tPex20<-PpPchB_Yl<-PrGPD::PrTEFin->EcEntC_Yl->tLip2 | Plasmid carrying <-PrGPD::PrTEFin-> for amplification |  |  | [1] |
| **Basic integrative vectors** | | | | | |
| pCfB6367 | E-1 | Backbone plasmid for construction of gene integration plasmids at E-1 site |  |  | [2] |
| pCfB6679 | E-4 | Backbone plasmid for construction of gene integration plasmids at E-4 site |  |  | [2] |
| **gRNA vectors** | | | | | |
| pCfB6633 | pNat-YLgRNA2_IntE_1 | Plasmid carrying gRNA to cut at E-1 site |  |  | [2] |
| pCfB6638 | pNat-YLgRNA2_IntE_4 | Plasmid carrying gRNA to cut at E-4 site |  |  | [2] |
| **Integrative vectors** | | | | | |
| pCfB9216 | E-1-CpEgt2-Yl<-PrGPD::PrTEFin->NcEgt1 | Integration of CpEgt2-<-PrGPD::PrTEFin->NcEgt1 at E-1 site | pCfB6367 | BB3907  BB4204, BB4205 | This study |
| pCfB9324 | E-1-NcEgt1-Yl<-PrGPD::PrTEFin->CpEgt2) | Integration of NcEgt1<-PrGPD::PrTEFin->CpEgt2 at E-1 site | pCfB6367 | BB3907,  BB4332,  BB4333 | This study |
| pCfB10020 | E_4-NcEgt1-<-PrGPD-TEFin->CpEgt2 | Integration of CpEgt2-<-PrGPD::PrTEFin->NcEgt1 at E-4 site | pCfB6679 | BB3907,  BB4204,  BB4205 | This study |

**Table S6:** List of strains used in this study.

| **Strain** | **Characteristics** | **Strain specifics** | **Parent strain** | **Genetic edit** | **Source** |
| --- | --- | --- | --- | --- | --- |
| ST6512 | *Y. lipolytica* W29 MATA ku70∆::PrTEF1-Cas9-TTef12::PrGPD-DsdA-TLip2 | Background strain for *Yarrowia* *lipolytica* strains, Cas9 integrated into the genome, deletion of *ku70* for decreased non-homologous end-joining |  |  | [2] |
| ST9584 | CpEgt2<-PrGPD::PrTEFin->NcEgt1 | Fungal pathway under different strong promoters | ST6512 | pCfB6633,  pCfB9216 | This study |
| ST9703 | NcEgt1<-PrGPD::PrTEFin->CpEgt2 | Fungal pathway under different strong promoters | ST6512 | pCfB6633,  pCfB9324 | This study |
| ST10264 | NcEgt1<-PrGPD::PrTEFin->CpEgt2 + CpEgt2<-PrGPD::PrTEFin->NcEgt1 | Two copies of fungal pathway both expressed under two different strong promoters | ST9584 | pCfB6638,  pCfB10020 | This study |

**References:**

1 Sáez-Sáez J, Wang G, Marella ER, Sudarsan S, Cernuda Pastor M & Borodina I (2020) Engineering the oleaginous yeast Yarrowia lipolytica for high-level resveratrol production. *Metab Eng* **62**, 51–61.

2 Holkenbrink C, Dam MI, Kildegaard KR, Beder J, Dahlin J, Doménech Belda D & Borodina I (2018) EasyCloneYALI: CRISPR/Cas9-Based Synthetic Toolbox for Engineering of the Yeast Yarrowia lipolytica. *Biotechnol J* **13**, 1700543.
